# *ZmFAR1* and *ZmABCG26* Regulated by microRNA Are Essential for Lipid Metabolism in Maize Anther

**DOI:** 10.3390/ijms22157916

**Published:** 2021-07-24

**Authors:** Yilin Jiang, Ziwen Li, Xinze Liu, Taotao Zhu, Ke Xie, Quancan Hou, Tingwei Yan, Canfang Niu, Shaowei Zhang, Mengbing Yang, Rongrong Xie, Jing Wang, Jinping Li, Xueli An, Xiangyuan Wan

**Affiliations:** 1 Zhongzhi International Institute of Agricultural Biosciences, Shunde Graduate School, Research Center of Biology and Agriculture, University of Science and Technology Beijing (USTB), Beijing 100024, China; b20190393@xs.ustb.edu.cn (Y.J.); liziwen@ustb.edu.cn (Z.L.); b20180388@xs.ustb.edu.cn (X.L.); b20170369@xs.ustb.edu.cn (T.Z.); xieke@ustb.edu.cn (K.X.); houquancan@ustb.edu.cn (Q.H.); b20190395@xs.ustb.edu.cn (T.Y.); niucanfang@163.com (C.N.); b20200413@xs.ustb.edu.cn (S.Z.); yangmengbing1128@163.com (M.Y.); s20200902@xs.ustb.edu.cn (R.X.); 18810576992@163.com (J.W.); 2 Beijing Engineering Laboratory of Main Crop Bio-Tech Breeding, Beijing International Science and Technology Cooperation Base of Bio-Tech Breeding, Beijing Solidwill Sci-Tech Co. Ltd., Beijing 100192, China; lijinping@sjlhtech.com

**Keywords:** noncoding RNA, microRNA, lipid metabolism, anther and pollen development, genic male sterility, maize (*Zea mays*)

## Abstract

The function and regulation of lipid metabolic genes are essential for plant male reproduction. However, expression regulation of lipid metabolic genic male sterility (GMS) genes by noncoding RNAs is largely unclear. Here, we systematically predicted the microRNA regulators of 34 maize white brown complex members in ATP-binding cassette transporter G subfamily (WBC/ABCG) genes using transcriptome analysis. Results indicate that the *ZmABCG26* transcript was predicted to be targeted by zma-miR164h-5p, and their expression levels were negatively correlated in maize B73 and Oh43 genetic backgrounds based on both transcriptome data and qRT-PCR experiments. CRISPR/Cas9-induced gene mutagenesis was performed on *ZmABCG26* and another lipid metabolic gene, *ZmFAR1*. DNA sequencing, phenotypic, and cytological observations demonstrated that both *ZmABCG26* and *ZmFAR1* are GMS genes in maize. Notably, ZmABCG26 proteins are localized in the endoplasmic reticulum (ER), chloroplast/plastid, and plasma membrane. Furthermore, ZmFAR1 shows catalytic activities to three CoA substrates in vitro with the activity order of C12:0-CoA > C16:0-CoA > C18:0-CoA, and its four key amino acid sites were critical to its catalytic activities. Lipidomics analysis revealed decreased cutin amounts and increased wax contents in anthers of both *zmabcg26* and *zmfar1* GMS mutants. A more detailed analysis exhibited differential changes in 54 monomer contents between wild type and mutants, as well as between *zmabcg26* and *zmfar1*. These findings will promote a deeper understanding of miRNA-regulated lipid metabolic genes and the functional diversity of lipid metabolic genes, contributing to lipid biosynthesis in maize anthers. Additionally, cosegregating molecular markers for *ZmABCG26* and *ZmFAR1* were developed to facilitate the breeding of male sterile lines.

## 1. Introduction

Anther and pollen development are critical for male reproduction of flowering plants. Plant microspore mother cells are surrounded by an anther wall consisting of four layers, from outer to inner including epidermis, endothecium, middle layer, and tapetum [1]. Specifically, developing pollen grains are protected from external abiotic stresses and biotic attacks by the anther cuticle that is an extracellular lipidic layer covering anther’s outer surface. Additionally, the pollen wall, which consists of multiple layers including pollen outer surface (exine), protects the male gametophytes from various environmental stresses and plays a crucial role in pollen–stigma interaction and fertilization [2,3]. The development of anther cuticle and pollen wall is closely associated with lipid metabolism, especially lipid biosynthesis and transport in plant anthers [4]. Lipid metabolism is also important for pollen maturation, anther dehiscence, and membrane system formation of subcellular organelles in the different anther wall layers [3,4,5,6,7]. Therefore, lipid metabolism has both structural and functional significance for plant male reproduction.

Small noncoding RNAs, such as microRNAs (miRNAs), have expression-regulatory functions on target genes at the posttranscriptional and/or translational levels in plants and animals and are important regulators of plant growth and reproduction processes, and response to stress. In plants, several miRNAs have been reported as important regulators contributing to anther development and pollen formation by targeting transcription factor (TF) genes, such as miR164-*NAC* (NAM, ATAF, and CUC) module controlling stamen development [8], miR159-*GAMYB* module regulating anther development and stress response [9], miR319-*TCP* (teosinte-branched 1/cycloidea/proliferating) module modulating secondary cell wall thickening of anther endothecium cells [10], and miR167-*ARF* (auxin-responsive factor) module regulating anther dehiscence [11], as well as other miRNA-TF gene modules involved in male fertility [12]. Though the targets of miRNAs are frequently identified as TF genes, miRNAs also bind and fine-tune the expression of non-TF genes to control plant male reproduction. For example, miR528 controlled pollen intine formation by targeting a blue copper protein gene [13], and miR399 influenced anther dehiscence and pollen fertility by targeting an ubiquitin-conjugating E2 enzyme gene [14]. Nevertheless, metabolic enzyme or transporter genes contributing to anther development by miRNA-mediated gene expression were rare. Therefore, the posttranscriptional regulations of metabolic genes by miRNAs and their roles in anther development need to be further revealed and investigated.

Plants have a large family of ATP-binding cassette (ABC) transporters, and the ABCG transporter is the largest subfamily in *Arabidopsis*, rice, and maize [15,16]. ABCG transporters are important for the transportation of wax and cutin monomers [17]. Many ABCG transporters were shown to be essential for anther and pollen development. In *Arabidopsis*, the *ATP-binding cassette subfamily G26* (*AtABCG26*) encoded a key transporter protein for pollen exine formation by transferring lipid precursors and polyketides [18,19]. Similarly, *AtABCG1*, *AtABCG9*, *AtABCG16*, and *AtABCG31* were found to be related to pollen wall formation [20,21]. *AtABCG13* was reported to be highly expressed in flowers and required for the transport of cuticular lipids [22]. AtABCG11 protein specifically transported cutin and wax precursors from the epidermis to the anther outer surface [23,24]. AtABCG11 could also form homo- or heterodimers with AtABCG12 to transport different cuticular components [23]. In *Medicago truncatula*, *SGE1* (*stigma exsertion1*) encoded an ABCG transporter that played a critical role in regulating floral cutin and wax secretion and physically interacted by a heterodimer form with another half-size transporter, MtABCG13 [25]. In rice, OsABCG15 and OsABCG26 (orthologs of AtABCG26 and AtABCG11, respectively) were important for lipid transport during anther and pollen development [26,27]. *OsABCG3/LSP1* encoded a half-size ABCG transporter, and its loss-function caused abnormal degradation of tapetum and a lack of nexine II and intine layers development [28,29]. In maize, *ZmGL13* encoded a putative ABCG protein that showed involvement in the transport of epicuticular waxes onto the surfaces of seedling leaves [30]. Maize *Male sterility 2* (*ZmMs2*) encoded an ABCG transporter and may function in lipidic molecule transport [31]. The functions and expression regulations of ABCG family members, especially the posttranscriptional regulation by miRNAs, require further research.

Fatty acyl reductases (FARs) catalyze the NADPH-dependent reaction for the conversion of fatty acyl-coenzyme A (CoA) or acyl carrier protein (ACP) to a primary fatty alcohol. Fatty alcohols and their derivatives are major components of the lipidic anther cuticle and pollen wall [32]. In *Arabidopsis*, *AtMs2/AtFAR2* encoded a fatty acid reductase that functioned in the production of fatty alcohols in plastids [33,34], and its expression was directly regulated by AtMYB103/AtMS188/AtMYB80 [35]. The gene *AtFAR3*/*CER4* encoded an endoplasmic reticulum (ER)-localized FAR that was specifically involved in the production of C24 to C28 very long-chain primary alcohols [36]. In rice, *Defective Pollen Wall* (*OsDPW*), the ortholog of *AtMs2*, was similarly regulated by OsMYB80 and controlled the primary fatty alcohol synthesis for the anther cuticle and sporopollenin [37,38]. In bread wheat (*Triticum aestivum* L.), TaTAA1 encoded by *Triticum aestivum anther 1* was the first reported alcohol-forming fatty acyl–CoA reductase responsible for male gametophyte development [39]. In maize, *ZmMs6021/ZmMs25* [40,41] encoded a fatty acyl–ACP reductase and was directly activated and regulated by ZmMYB84, which is an MYB TF ortholog of AtMYB80 and OsMYB80 [41]. Even though the major catalytic steps of FARs in lipid biosynthesis have been revealed, our knowledge on the function and regulation of FAR family members involved in plant male reproduction is still limited.

The present study focuses on the potential posttranscriptional regulation of *ZmABCG26* by zma-miR164h-5p during maize anther development and evaluation of *ZmABCG26* and *ZmFAR1* as genic male sterility (GMS) genes using the CRISPR/Cas9 mutagenesis to generate mutants for phenotypic and cytological observations. The subcellular localization of the two GMS genes encoding proteins was investigated, and analysis of enzymatic activity for ZmFAR1was conducted in vitro. Finally, a comparison was conducted for lipidomics data from anthers of *ZmABCG26* and *ZmFAR1* knockout lines. These findings will facilitate a greater understanding of the miRNA-regulated lipid metabolic network in maize anthers and may promote the application of lipid metabolic GMS genes in molecular breeding and hybrid seed production in maize.

## 2. Results

### 2.1. The Potential Posttranscriptional Regulation of ZmABCG26 by zma-miR164h-5p during Maize Anther Development

**Figure 1:**
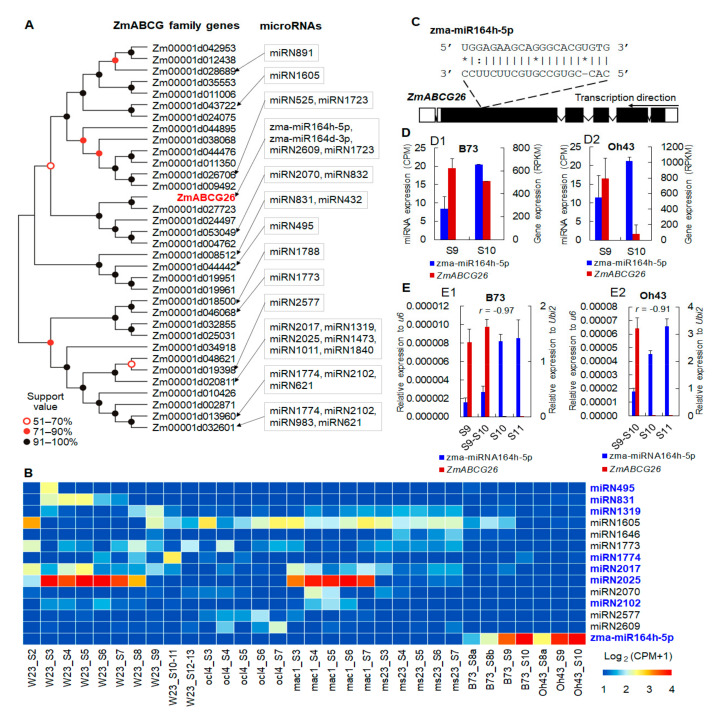
Posttranscriptional regulation of *ZmABCG26* by zma-miR164h-5p during maize anther development: (**A**) the phylogenetic tree of maize ABCG family genes and their predicted miRNA regulators during anther development; (**B**) transcriptome analyses of expression patterns of predicted miRNA regulators of maize WBC/ABCG family genes in anthers of WT inbred lines (W23, B73, and Oh43) and GMS mutant lines (*ocl4*, *mac1*, *ms23*). The names of predicted miRNA regulators having negatively correlated expression levels with their ABCG target genes (Pearson correlation test) were marked in blue; (**C**) the sequence alignment of zma-miR64h-5p and its target site on the *ZmABCG26* transcript. *, base mismatch; (**D**,**E**) the negatively correlated expression patterns between *ZmABCG26* and zma-miR164h-5p in B73 (**D1**,**E1**) and Oh43 (**D2**,**E2**) inbred lines at anther developmental stages 9 and 10 revealed by transcriptome analyses (**D**) and at stages 9 to 11 confirmed by qRT-PCR analyses (**E**). *r*, Pearson correlation coefficient.

*ZmABCG26* has been predicted as one of the targets of zma-miR164h-5p by bioinformatics analysis based on transcriptomes of maize anthers [42]. *ZmABCG26* belongs to the WBC/ABCG subfamily that contains 34 members in the maize genome (Figure 1A). To further investigate the potential posttranscriptional regulation of *ZmABCG26* by zma-miR164h-5p during maize anther development, we predicted the miRNA regulators of all maize WBC/ABCG family genes with additional transcriptome datasets. By a combined analysis of RNA-Seq and miRNA-Seq data from three maize WT lines (W23, Oh43, and B73), as well as three maize GMS mutants (*mac1*, *ocl4*, and *ms23*) [12,42,43], 29 miRNA-ABCG gene pairs were predicted that contained 13 maize WBC/ABCG family members and a total of 2 known and 23 potential new miRNAs (Figure 1A, Table S1). Among the 25 miRNAs, 13 have detectable expression values in the anther transcriptome dataset (Figure 1B). zma-miR164h-5p was highly expressed in B73 and Oh43 during anther developmental stages 8 to 9. miRN2025 was also highly expressed during anther developmental stages 3 to 7 in W23 and mutant *ocl4*. In addition, 8 of the 13 expressed known or potential miRNAs were negatively correlated with their target genes in expression levels, including zma-miR164h-5p, which was predicted to bind to the *ZmABCG26* transcript at the region corresponding to the fifth exon (Figure 1C,D and Figure S1). In B73 anther transcriptomes, zma-miR164h-5p was increasingly expressed during anther developmental stages 9 to 10, and the expression level of *ZmABCG26* was decreased during the corresponding stages (Figure 1(D1)). Similarly, the negatively correlated expression pattern between zma-miR164h-5p and *ZmABCG26* was detected in the Oh43 anther transcriptomes during the same developmental stages (Figure 1(D2)). Furthermore, expression patterns between zma-miR164h-5p and *ZmABCG26* were confirmed by quantitative reverse transcription PCR (qRT-PCR) assay in B73 and Oh43 anthers during a broader and finely divided developmental period that includes stages 9, 9–10, 10 and 11 (Figure 1E). The qRT-PCR results showed the expression of zma-miR164h-5p was continually maintained at a high level in B73 or increased into a higher level in Oh43 at anther developmental stages 10 and 11 (Figure 1(E1,E2)), while the expression of *ZmABCG26* was hardly detected in the two lines at stages 10 and 11. Therefore, by the expression anticorrelation result, it can be speculated that *ZmABCG26* is most probably regulated by zma-miR164h-5p at the posttranscriptional level, and the miR164h-*ABCG26* module may be functionally important in maize anther development.

### 2.2. CRISPR/Cas9-Induced Loss Function of ZmABCG26 Leads to Complete Male Sterility in Maize

**Figure 2:**
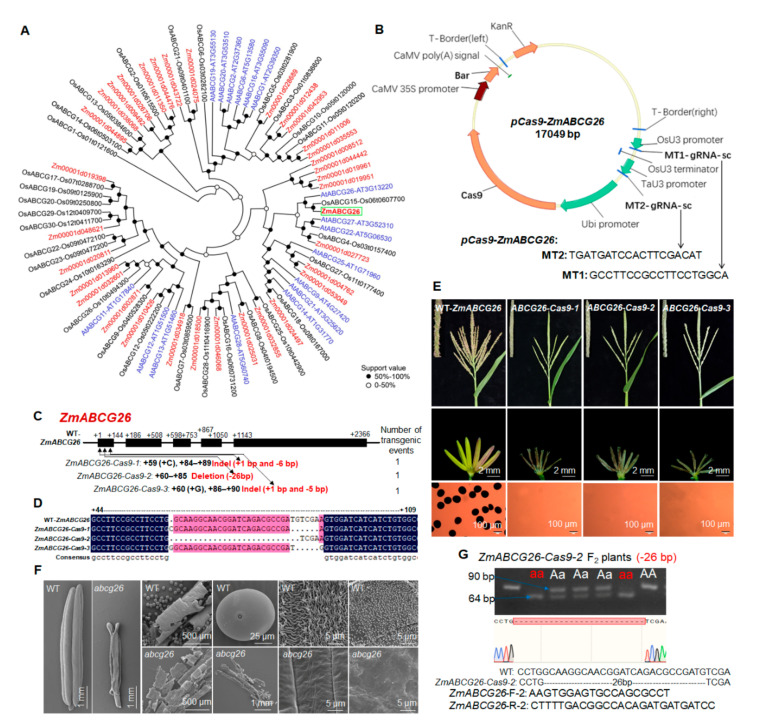
Phylogenetic tree of WBC/ABCG family members, phenotypic characterization, sequencing analysis, and cosegregating molecular marker development of CRISPR/Cas9-induced *ZmABCG26* male-sterile mutants: (**A**) phylogenetic tree of the WBC/ABCG family members in *Arabidopsis* (At), rice (Os), and maize (Zm). A neighbor-joining tree showed the evolutionary relationships of WBC/ABCG members in the three species; (**B**) physical map of *pCas9-ZmABCG26* construct carrying two gRNAs and the information of target sites in *ZmABCG26*; (**C**) gene structure of *ZmABCG26* and mutation analysis of three knock-out lines (*ZmABCG26-Cas9-1*, *-2*, and *-3*) created by the CRISPR/Cas9 technology; (**D**) DNA sequencing of targeted mutation sites or fragments among WT and the three knockout lines; (**E**) comparison of tassels, anthers, and pollen grains stained with I_2_-KI among WT and the three knockout lines; (**F**) SEM analysis of the whole anthers, pollen grains, anther outer and inner surfaces in WT and one *abcg26* mutant at anther developmental stage 13; (**G**) development of cosegregating molecular markers for genotyping *ZmABCG26-Cas9-2* mutation in F_2_ plants.

The phylogenetic analysis predicted that *ZmABCG26 (Zm00001d046537)* was an orthologous gene of GMS genes *OsABCG15* and *AtABCG26* (Figure 2A). Using the CRISPR/Cas9 technology *zmabcg26* mutants were generated to evaluate the functional importance of *ZmABCG26* in maize male reproduction. Firstly, a *pCas9-ZmABCG26* vector with two targets in the first exon of *ZmABCG26* was constructed (Figure 2B) and then transformed into maize. PCR products containing the target site fragment of primary T_0_ transgenic plants were sequenced and three homozygous loss-of-function mutants of *ZmABCG26* with 7-bp indel, 26-bp deletion, and 6-bp indel, respectively, were obtained (Figure 2C,D). The three *zmabcg26* homozygous mutants exhibited normal vegetative organ growth and female development but showed shrunken anthers that failed to produce viable pollen grain, as compared to WT plants (Figure 2E). Scanning electron microscopy (SEM) assay indicated that the outer and inner surfaces of *zmabcg26* mutant anther wall were smooth and glossy without knitting cuticle and Ubisch bodies (Figure 2F). These results indicated that *ZmABCG26* plays an essential role in maize anther and pollen development.

T_0_ plants did not produce seeds by self-pollination, and maize inbred line Zheng58 was used as a male parent to pollinate plants. To avoid the complications from the continuing action of the Cas9/gRNA transgene element in Zheng58 alleles, Cas9-negative F_1_ plants were selected to produce F_2_ seeds. To detect genotypes of the *zmabcg26* mutant F_2_ plants, the cosegregating molecular markers with the primer pairs covering the corresponding mutations in *ZmABCG26* were developed, by which the mutant genotypes were identified using PCR amplification, together with agarose gel electrophoresis or polyacrylamide gel electrophoresis (PAGE) (Figure 2G, Table S2).

### 2.3. Spatiotemporal Expression Analysis of ZmABCG26 Gene and Subcellular Localization of ZmABCG26 Protein

**Figure 3:**
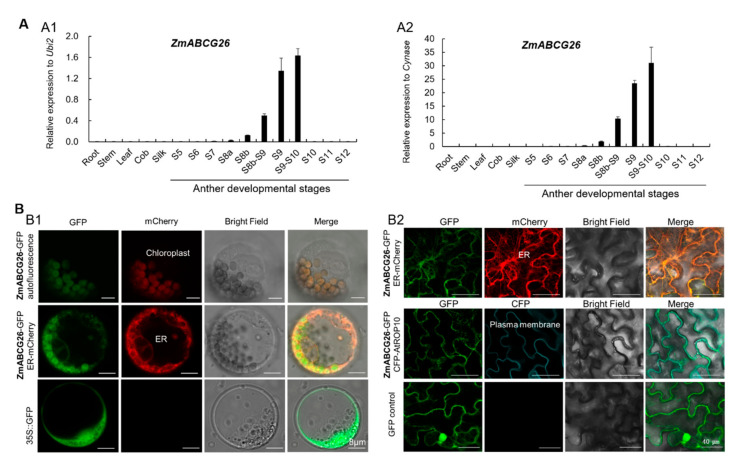
Spatiotemporal expression analysis of *ZmABCG26* gene and subcellular localization of ZmABCG26 protein: (**A**) spatiotemporal expression analysis of *ZmABCG26* by qRT-PCR assay with *ZmUbi2* (**A1**) and *ZmCynase* (**A2**) as the internal control, respectively. Error bars indicate SD. Each reaction was performed in three biological replicates with three technical repeats; (**B**) confocal images showing the subcellular localization of ZmABCG26 in maize protoplasts and tobacco leaf cells. The constructs of *ZmABCG26-GFP*, *35S-GFP*, *ER-mCherry* (*mCherry-HDEL*), and *CFP-AtROP10* were expressed in maize protoplasts (**B1**) or tobacco leaf cells (**B2**). The chlorophyll autofluorescence was used as a plastid marker. The *ZmABCG26-GFP* was cotransformed with the *ER-mCherry* as an ER marker in maize protoplasts and tobacco leaf cells, and also cotransformed with the *CFP-AtROP10* as a plasma membrane marker in tobacco leaf cells. The *35S-GFP* vector was used as a negative control. Scale bars, 8 µm (**B1**) and 40 µm (**B2**).

*ZmABCG26* was expressed in anthers from stages 8b to 9–10, with the expression peak at stage 9–10, while its expression was not detected in the vegetative tissues (root, stem, and leaf) and female organs (immature cob and silk) (Figure 3A), indicating that *ZmABCG26* is an anther-specific expression GMS gene. The expression pattern of *ZmABCG26* is consistent with its functions in maize anther and pollen development.

Transiently expressed *ZmABCG26-GFP* constructs in maize protoplasts and tobacco leaves, respectively, were used to investigate the subcellular localization of ZmABCG26. In maize protoplasts, the ZmABCG26-GFP fusion protein was colocalized with both autofluorescence of chlorophyll in plastids and the ER marker protein mCherry-HDEL (Figure 3(B1)). In tobacco leaf epidermis, ZmABCG26-GFP fusion protein was colocalized with both the ER marker and plasma membrane marker proteins (Figure 3(B2)). Therefore, ZmABCG26 is primarily localized in ER, plasma membrane, and chloroplasts/plastids, implying that the functions of ZmABCG26 and its orthologs may be slightly different between plant species.

### 2.4. CRISPR/Cas9-Based Confirmation of ZmFAR1 Function in Controlling Maize Male Sterility

**Figure 4:**
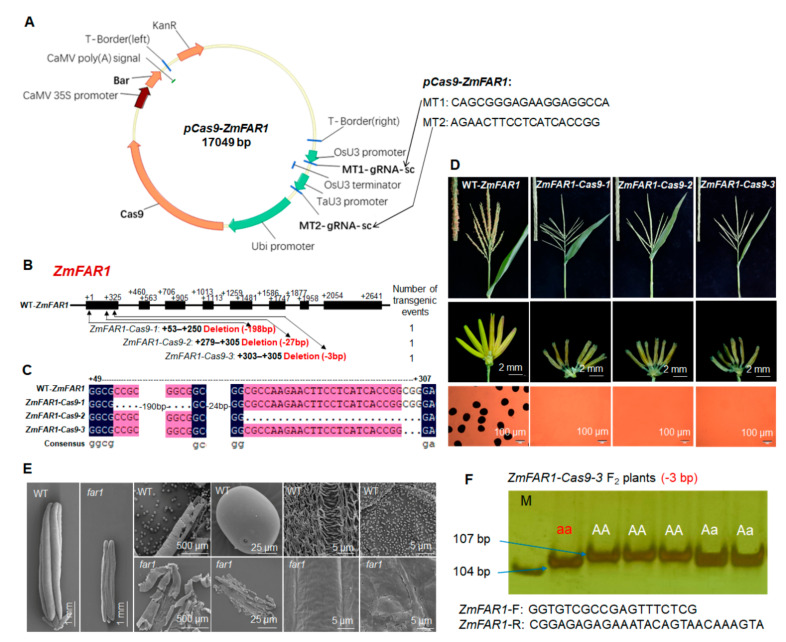
Phenotypic characterization, sequencing analysis, and cosegregating molecular marker development of CRISPR/Cas9-induced *ZmFAR1* male-sterile mutants: (**A**) physical map of *pCas9-ZmFAR1* construct carrying two gRNAs and the information of target sites in *ZmFAR1*; (**B**) gene structure of *ZmFAR1* and mutation analysis of three knock-out lines (*ZmFAR1-Cas9-1*, *-2* and *-3*) created by the CRISPR/Cas9 technology; (**C**) DNA sequencing of targeted mutation sites or fragments among WT and the three knockout lines; (**D**) comparison of tassels, anthers, and pollen grains stained with I_2_-KI among WT and the three knockout lines; (**E**) SEM analysis of whole anthers, pollen grains, anther outer and inner surfaces in WT, and one *zmfar1* mutant at anther developmental stage 13; (**F**) development of cosegregating molecular marker for genotyping *ZmFAR1-Cas9-3* mutation in F_2_ plants.

*ZmFAR1* (*GRMZM2G120987/Zm00001d048337*) is the maize ortholog of GMS genes *OsDPW* and *AtMs2* encoding fatty acyl reductases (FARs) [34,37]. One CRISPR/Cas9 vector of *ZmFAR1* was constructed and transformed into maize (Figure 4A). Three homozygous *zmfar1* mutants (*ZmFAR1-Cas9-1*, *-2* and *-3*) with 198-bp, 27-bp, and 3-bp deletions in the first exon, respectively (Figure 4B,C), were produced for the evaluation. *zmfar1* mutant F_2_ plants exhibited normal vegetative organ growth and female fertility but showed complete male sterility with smaller anthers lacking pollen grains, compared with WT (Figure 4D). SEM assay revealed *zmfar1* mutant anthers lacked the knitting cuticle and Ubisch bodies (Figure 4E). These results confirmed that *ZmFAR1* is indispensable to male fertility in maize. Cosegregating molecular markers were developed and successfully used to detect *zmfar1* mutant genotypes via PCR amplification with gel electrophoresis (Figure 4F, Table S2).

### 2.5. Spatiotemporal Expression Analysis of ZmFAR1 Gene and Subcellular Localization and Enzymatic Activity Analysis of ZmFAR1 Protein

**Figure 5:**
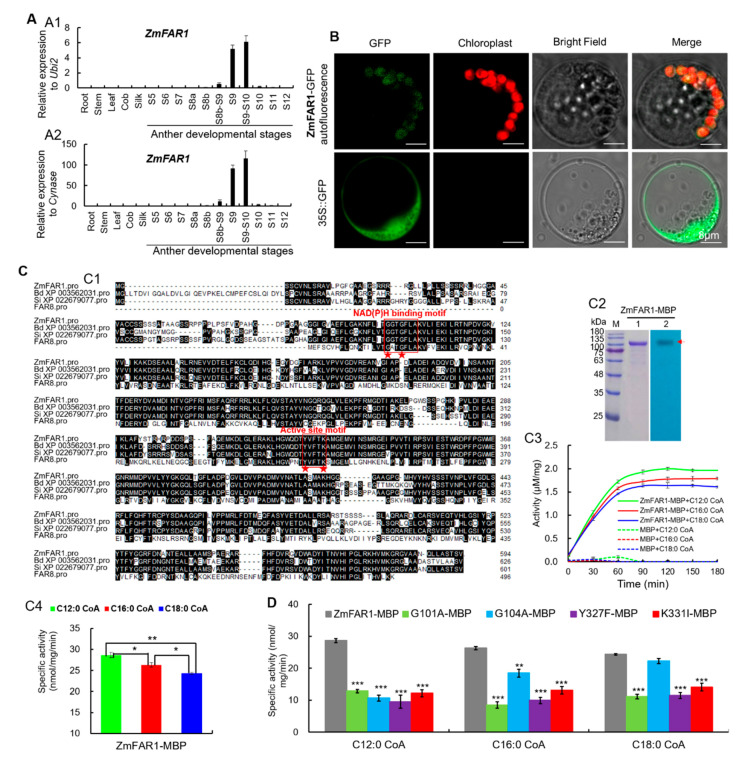
Spatiotemporal expression analysis of *ZmFAR1* and subcellular localization and enzyme activity analysis of ZmFAR1 protein: (**A**) spatiotemporal expression analysis of *ZmFAR1* by qRT-PCR assay with *ZmUbi2* (**A1**) and *ZmCynase* (**A2**) as the internal controls, respectively. Error bars indicate SD. Each reaction was performed in three biological replicates with three technical repeats: (**B**) confocal images showing the subcellular localization of ZmFAR1 in maize protoplasts. The constructs of *ZmFAR1-GFP* and *35S-GFP* were expressed in maize protoplasts. The chlorophyll autofluorescence was used as a plastid marker. The *35S-GFP* vector was used as a negative control; (**C**) ZmFAR1 possessed catalytic activities to three fatty acyl–CoA substrates in vitro: (**C1**) alignment of ZmFAR1 and its putative orthologs in plants based on the CLUSTALW program. The conserved NAD(P)H binding Rossmann-fold domain (GGTGFLA) and active site motif (YVFTK) in the FAR family were highlighted with the red rectangular borders. The red pentagrams indicated the key amino acids in the GGTGFLA motif for NAD(P)H binding and in the predicted active site motif; (**C2**) prokaryotic expression and Western blotting analysis of the MBP-tagged protein ZmFAR1 (ZmFAR1-MBP) purified from *E. coli*. Line 1, SD–SPAGE; line 2, Western blotting; (**C3**,**C4**) ZmFAR1-MBP showed different catalytic activities to three substrates C12:0-, C16:0-, and C18:0-CoAs in vitro (**C3**) with the activity order of C12:0-CoA > C16:0-CoA > C18:0-CoA (**C4**). Zm, *Zea mays*; Bd, *Brachypodium distachyon*, and Si, *Setaria italica*. * and ** indicated the significant levels of 5% and 1% (Student’s *t* test, *n* = 3), respectively; (**D**) four amino acid mutations (G101A, G104A, Y327F, and K331I) of ZmFAR1-MBP showed a significant reduction of enzymatic activities against all three substrates C12:0-, C16:0-, and C18:0-CoAs *in vitro*, compared to the WT ZmFAR1-MBP except for G104A against C18:0-CoA. Notes: ** and *** indicated the significant levels of 1% and 1‰ (Student’s *t* test, *n* = 3), respectively.

qRT-PCR analysis showed *ZmFAR1* expression was undetectable in vegetative organs (root, stem, and leaf) and female organs (immature cob and silk), but was specific in anthers from stages 8b-9 to 9–10 with the peak at stage 9–10 (Figure 5A). To determine the subcellular localization of ZmFAR1 in maize, the *ZmFAR1-GFP* construct was transiently expressed in maize protoplasts, and ZmFAR1-GFP fusion protein was colocalized with autofluorescence of chlorophyll in plastids (Figure 5B), indicating that ZmFAR1 functions as a plastid-localized fatty acyl reductase.

Enzymatic activity analysis of ZmFAR1 was based on the prokaryotic expression system and in vitro activity assay. ZmFAR1 contains a NAD(P)H-binding Rossmann-fold domain with two conserved motifs GGTGFLA and YVFTK at the N terminus and a FAR_C domain at the C terminus. Alignment of ZmFAR1 and its putative orthologs in plants revealed that there were four conserved residues, i.e., G101 and G104 in motif I (GGTGFLA) and Y327 and K331 in motif II (YVFTK) (Figure 5(C1)). The MBP-tagged ZmFAR1 protein (ZmFAR1-MBP) was expressed in *Escherichia coli* and confirmed by Western blotting (Figure 5(C2)). Then, using three fatty acyl–CoAs (C12:0-, C16:0- and C18:0-CoAs) as substrates, the enzymatic activities of ZmFAR1 were investigated in vitro. Based on a spectrophotometric assay following the consumption of NADPH, ZmFAR1 catalyzed the reduction of the three fatty acyl–CoAs (Figure 5(C3)) and showed significantly higher catalytic activity to C12:0-CoA than C16:0- and C18:0-CoAs (Figure 5(C4)), suggesting that ZmFAR1 has enzyme activities to multiple fatty acyl–CoA substrates with various chain lengths (C12 to C18). In addition, the effects of four mutations (G101A, G104A, Y327F, and K331I) on substrate activities were also evaluated and compared with WT ZmFAR1. Almost all the four variant enzymes showed significantly decreased specific activities to the three fatty acyl–CoAs (Figure 5D), indicating that all the substituted residues at positions 101, 104, 327, and 331 can significantly impair the reduction capability of ZmFAR1 toward different substrates.

### 2.6. Functional Comparison of ZmABCG26 and ZmFAR1 Contributing to Lipid Metabolism in Maize Anthers

The cytological defects of anther cuticles (Figure 2FandFigure 4E) indicated that *zmabcg26* and *zmfar1* mutations may alter lipid biosynthesis or transport for anther cuticle formation. Anthers of WT and two mutants collected before anthesis were used to detect the components of cutin, wax, and internal lipid by using gas chromatography–mass spectrometry (GC–MS).

**Figure 6:**
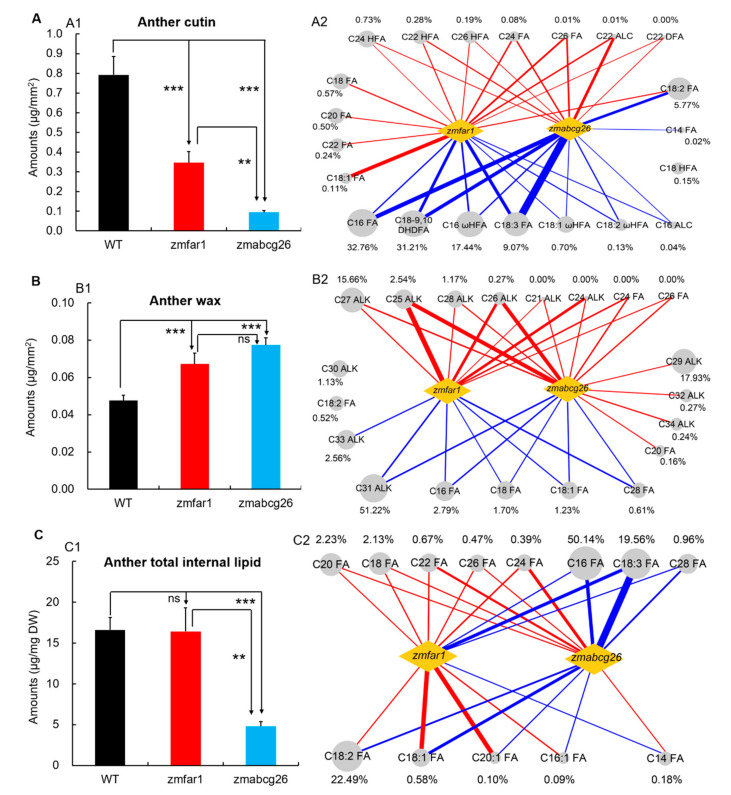
Comparison of genes *ZmFAR1* and *ZmABCG26* for lipid metabolism of maize anthers by lipidomics analysis: (**A**) the amount of anther cutin in WT and mutants *zmfar1* and *zmabcg26* at stage 13: (**A1**) the total amount of anther cutin per unit surface area; (**A2**) the changes of 21 specific cutin monomers in *zmfar1* and *zmabcg26* anthers, compared to those in the WT anther at stage 13; (**B**) the amount of anther wax in WT and mutants *zmfar1* and *zmabcg26* at stage 13: (**B1**) the total amount of wax per unit surface area; (**B2**) the change range of 20 specific wax constituents in *zmfar1* and *zmabcg26* anthers at stage 13, compared to those in WT; (**C**) the number of internal lipid constituents in WT and mutants *zmfar1* and *zmabcg26* at stage 13: (**C1**) the total amount of internal lipid constituents per unit dry weight; (**C2**) the change range of 13 specific internal lipid constituents in *zmfar1* and *zmabcg26* anthers at stage 13, compared to those in WT. In (**A1**,**B1**,**C1**), ** and *** indicate the significant levels of 1% and 1‰ (Student’s *t* test, *n* = 3), respectively. In (**A2**,**B2**,**C2**), the red or blue lines represent the increase or decrease of the cutin, wax, and internal lipid monomers in mutant anthers, respectively. The thickness of the lines represents the change magnitude of the increase or decrease.

Lipidomics analysis showed that the total anther cutin contents in *zmfar1* (0.345 µg/mm^2^) and *zmabcg26* (0.095 µg/mm^2^) were significantly lower (*p* < 0.001) than that in WT (0.793 µg/mm^2^), and the total cutin content in *zmabcg26* anther was significantly lower (*p* < 0.01) than that in *zmfar1* anther (Figure 6A(1)), indicating *zmabcg26* mutation had a stronger effect on the reduction of anther cutin content. The differences of total cutin content among WT, *zmfar1*, and *zmabcg26* anthers were mainly due to the alterations of 21 cutin monomers, including seven reduced (91.35% of the total cutin), seven increased (1.29% of the total cutin), and seven oppositely or differently changed (7.36% of the total cutin) (Figure 6A(2), Figure S2). Notably, the content of C18:2 FA (5.57% of the total cutin) was significantly increased in *zmfar1* anther but decreased in *zmabcg26* anther.

The total wax contents in *zmfar1* and *zmabcg26* anthers (0.067 and 0.077 µg/mm^2^, respectively) were significantly higher (*p* < 0.001) than that (0.048 µg /mm^2^) in WT anther (Figure 6(B1)). Although the total wax content of *zmabcg26* was seemingly higher than that of *zmfar1*, the difference was not statistically significant. Eight wax constituents, C21 and C24–28 alkanes (ALKs) (19.64% of the total wax) and C24 and C28 FAs (undetectable in WT), were greatly increased in both *zmfar1* and *zmabcg26* anthers when compared to those in WT anther, which resulted in significantly higher contents of total wax in the two mutant anthers (Figure 6(B2), Figure S3). Interestingly, some of the wax monomers displayed different alterations between *zmfar1* and *zmabcg26* anthers. For example, the amounts of C29, C32, and C34 ALKs (18.44% of the total wax) and C20 FA content (0.16% of the total wax) were significantly increased only in *zmabcg26* anther, while C33 ALK amount (2.56% of the total wax) was significantly reduced only in *zmfar1* anther (Figure 6(B2) and Figure S3).

In addition, no significant difference in the total internal lipid contents was found between *zmfar1* (16.44 µg/mg DW) and WT anthers (16.62 µg/mg DW), while the total internal lipid in *zmabcg26* anther (4.81 µg/mg DW) was greatly decreased when compared with those in WT and *zmfar1* anthers (Figure 6(C1)), suggesting that *ZmFAR1* and *ZmABCG26* play significantly different roles in the metabolism of internal lipid in maize anthers. Notably, the contents of four internal lipid constituents (C16:1, C18:1, C18:2, and C20 FAs, 23.26% of the total internal lipid) were significantly increased in *zmfar1* anthers but significantly decreased in *zmabcg26* anthers. Inversely, the level of C14 FA (0.18% of the total internal lipid) was significantly decreased in *zmfar1* anthers but significantly increased in *zmabcg26* anthers (Figure 6(C2) and Figure S4).

Therefore, lipidomics analysis indicates that both ZmABCG26 and ZmFAR1 participate in anther lipid metabolism that is necessary for anther and pollen development in maize, but they play significantly different roles in the metabolism of cutin, wax, and internal lipid in maize anthers.

### 2.7. ZmFAR1 and ZmABCG26 May Be Regulated by a Complex TF Regulatory Network during Anther Development

In addition to miRNAs, the expressions of lipid metabolic GMS genes are usually regulated by TF GMS genes, and also by feedback regulation of other lipid metabolic GMS genes. The expression pattern changes of *ZmFAR1* and *ZmABCG26* in anther development were analyzed by using seven sets of maize anther transcriptomes, including three TF GMS mutant lines (*ms23*, *ms7-6007*, and *lob30*) [12,42,44,45], two TF overexpression GMS lines (*p5126-ZmMs7* and *p5126-ZmLOB30*) [44], and two lipid metabolic GMS mutant lines (*ms30* and *ms33*) [7,46,47,48] (Figure S5A,B, Table S3). In *ms23* mutant, *ZmFAR1* expression was not significantly changed, while *ZmABCG26* expression was significantly downregulated at stages 6 and 7 (Figure S5(A1), indicating TF ZmMs23 may positively regulate *ZmABCG26* expression. The expression of *ZmFAR1* displayed downregulation in *ms7-6007* mutant and upregulation in *p5126-ZmMs7* overexpression line, while that of *ZmABCG26* was downregulated in *ms7-6007* mutant and its overexpression line (*p5126- ZmMs7*) (Figure S5(A2),(A3)), reflecting that TF ZmMs7 may positively regulate *ZmFAR1* and can affect *ZmABCG26* expression. The expressions of Z*mFAR1* and *ZmABCG26* were upregulated in *lob30* mutant, but downregulated in *p5126-ZmLOB30* overexpression line (Figure S5(A4),(A5)), suggesting that TF ZmLOB30 may negatively regulate Z*mFAR1* and *ZmABCG26*. *ZmFAR1* and *ZmABCG26* were not differentially expressed between WT and *ms30* or *ms33* mutants, showing that their expressions are not affected by lipid metabolic genes *ZmMs30* and *ZmMs33* (Figure S5B). These different or consistent expression pattern changes indicate that *ZmFAR1* and *ZmABCG26* may be regulated by a complex TF regulatory network.

The expression level of *ZmABCG26* was significantly changed in *zmfar1* anthers, compared with WT anthers at stages 8b and 9 (Figure S5C), implying the lipid biosynthetic gene *ZmFAR1* may affect the expression and function of the lipid transport gene *ZmABCG26* during anther development.

## 3. Discussion

### 3.1. miRNA-Mediated Lipid Metabolism Is Most Probably Critical for Maize Anther Development

In maize, though a certain number of miRNA regulatory modules and networks have been revealed by bioinformatics analyses [12,42], further experimental studies are required to confirm that the important roles of miRNAs have on regulating the expression of lipid metabolic genes during anther development. The negatively correlated expression pattern between zma-miR164h-5p and *ZmABCG26*, a lipid transporter gene, revealed in the present study, indicates the potential regulatory role of zma-miR164h-5p on *ZmABCG26* expression. *ZmABCG26* was demonstrated as a maize GMS gene by CRISPR/Cas9-mediated mutagenesis (Figure 2). These results suggest that the predicted miR164h-*ZmABCG26* module is most probably necessary for maize male reproductive process by regulating lipid transport, which supports the proposed miRNA-mediated regulatory module in anther lipid metabolism underlying plant male fertility. In addition to the known miRNA, several miRNA candidates were discovered by analyzing anther transcriptome data from different genetic backgrounds or mutant lines, and some of the miRNA candidates (e.g., miRN2025 in Figure 1B) have high expression levels and stage-specific expression patterns similar to those of the known miRNAs during anther development. Importantly, the miRNA candidates were predicted as potential expression regulators of ABCG family genes in maize (Figure 1A). This result implies that the newly predicted miRNA candidates may also contribute to anther development and pollen formation in maize. Nevertheless, the knockout/down and overexpression experiments of these miRNA loci as performed in some previous studies [11,13,14] should be required in future investigation to obtain more direct lines of evidence supporting the critical roles of the miRNA-ABCG gene modules for male reproduction in maize and other plant species.

### 3.2. ZmFAR1 and ZmABCG26 Display the Functional Differences in Anther Lipid Metabolism

Two lipidic barriers, anther cuticle and pollen exine covering the outer surfaces of anther and pollen grain, respectively, play important roles in protecting male reproductive development in flowering plants. The formation of anther cuticle and pollen exine shares a part of lipid metabolic pathways in the anther tapetum [4]. Accordingly, the tapetum is considered as the main place to produce cuticle and sporopollenin precursors that are transported to the anther or pollen surfaces for polymerization. Many tapetum-expressed lipid metabolic GMS genes have been reported to be involved in anther cuticle and pollen exine development, such as *ZmMs10*/*APV1* [49], *ZmMs20*/*IPE1* [50,51], *ZmMs26* [52], *ZmMs30* [46], and *ZmMs33* [47,48] in maize, and *OsCYP703A3* [53], *OsCYP704B2* [54], *OsDPW* [37], *OsABCG15* [26], and *OsABCG26* [27] in rice. In these GMS mutants, the lipidic contents and compositions of the anther cuticular layer are significantly altered, while comparing the impact of different lipid metabolic GMS genes on metabolic profiles of cutin and wax has been rarely reported.

Anther cuticle mainly consists of cuticular cutin and wax in which the lipidic polyester cutin lays on the anther surface, while the cuticular wax is embedded in the cutin matrix [55]. Cutin mainly consists of FA oxygenated derivatives with chain lengths of C16 and C18. In the present study, cutin amounts in *zmfar1* and *zmabcg26* anthers were significantly decreased (Figure 6A and Figure S2), which was similar to results reported in the mutants of their orthologous genes, *OsDPW* [37] and *OsABCG15* [26], indicating conserved roles in anther cutin synthesis and transport in monocot plants. Among the major cutin monomers, hydroxy FAs (C16 ωHFA and C18-9,10 DHDFA) and unhydroxylated FAs (C16 and C18 FAs) were significantly decreased in contents in *zmfar1* and *zmabcg26* anthers, and the change trends of these components were also observed in previously reported lipid metabolic mutants, such as *zmms30* [46], *zmms10/apv1* [49], and *zmms20/ipe1* [50,51], suggesting the functional conservation of these GMS genes in cutin synthesis and transport. Notably, the contents of five FAs (C18, C20, C22, C18:1, and C18:2 FAs) were increased only in *zmfar1* anther (Figure 6A and Figure S2), indicating functional divergences among lipid metabolic genes in controlling anther cuticle formation. In plant anthers, de novo FA (up to C18) synthesis occurs in plastids where *AtMs2* and *OsDPW*, the orthologs of *ZmFAR1*, function as FA reductases in the production of fatty alcohols. The content increases of C18, C18:1, and C18:2 FAs in *zmfar1* anthers may directly result from the deficient function of *ZmFAR1*, which leads to the inhibition of FA reduction in plastids.

Cuticular wax is a complex mixture of very long-chain FA derivatives [56], including ALKs, FAs, and fatty alcohols. The total wax contents in *zmfar1* and *zmabcg26* anthers were significantly increased (Figure 6B and Figure S3), which were predominantly due to significant increases in the contents of unsaturated alkanes, such as C24–C28, suggesting that the loss function of *ZmFAR1* and *ZmABCG26* may contribute positive feedback regulation of wax biosynthesis and transport. However, the amounts of C29, C32, and C34 alkanes were increased only in *zmabcg26* anthers, suggesting different positive feedback regulation mechanisms in *zmfar1* and *zmabcg26*. Similarly, a slight increase in the total amount of wax was also detected in rice *osdpw* anthers [37], reflecting the functional conservation.

ZmFAR1 and its orthologs, AtMs2 and OsDPW, are consistently localized in plastids and required for lipid biosynthesis in plant anthers. However, ZmABCG26 essential for lipid transport is primarily localized in ER, plasma membrane, and plastids (Figure 3B), which is not completely consistent with the subcellular localization of its orthologs, AtABCG26 and OsABCG15, which are localized on the plasma membrane [18,19,26], implying that the functions of ZmABCG26 and its orthologs may be slightly different between plant species.

The GMS mutants *zmabcg26* and *zmfar1* can be used for developing biotechnology-based male sterility (BMS) systems and creating male-sterile lines, which are critical for maize hybrid breeding and seed production. The cosegregating molecular markers developed based on CRISPR/Cas9-induced mutation sites or fragments in mutants *zmabcg26* and *zmfar1* (Figure 2G and Figure 4F) are beneficial to determine the mutant genotypes of crossing or backcrossing plants before flowering and pollination so that cross or backcross breeding can be efficiently performed to create male-sterile lines in different genetic backgrounds.

## 4. Materials and Methods

### 4.1. Plant Materials and Growth Conditions

Maize inbred lines B73, Oh43, and Zheng58 and hybrid Hi II were used in this study. B73 and Oh43 were originally obtained from the Maize Genetics Cooperation Stock Center (http://maizecoop.cropsci.uiuc.edu, accessed on 16 March 2011). Zheng58 and Hi II are maintained in our laboratory. All plants were grown at the experimental stations of the University of Sciences and Technology Beijing under normal cultivation conditions, T_0_ transgenic plants were grown in a greenhouse under long-day conditions (16 h/8 h (day/night) at 26 °C/22 °C).

### 4.2. Characterization of Mutant Phenotypes

Images of the tassels and anthers were captured with a Canon EOS 700D digital camera (Canon, Tokyo, Japan) and a SZX2-ILLB stereomicroscope (Olympus, Tokyo, Japan) respectively. Routine analysis of I_2_-KI staining and SEM (scanning electron microscopy) were performed as described previously [44]. SEM images of anther and pollen grain were detected with a HITACHI S-3400N scanning electron microscope (HITACHI, Tokyo, Japan).

### 4.3. RNA-Seq and Small RNA-Seq Analyses

The maize anther RNA-Seq and small RNA-Seq data of W23 line and *mac1*, *ocl4,* and *ms23* GMS mutant anthers were obtained based on a previous study [43]. The maize anther RNA-Seq and small RNA-Seq data of B73 and Oh43 lines were obtained from two previous studies [12,42]. RNA-Seq and small RNA-Seq data were analyzed according to the workflow of previous studies [12,42]. Raw data were processed and filtered by using NGSQCToolkit (v2.3.3) [57] for RNA-Seq data and Infernal program (v1.1.2) [58] and Rfam database (v14.0) [59] for small RNA-Seq data, and the remaining reads were mapped to the maize reference genome (AGPv4, Ensembl release 37) by using TopHat2 (v2.1.1) [60] for RNA-Seq data and Bowtie (v1.2.2) [61] for small RNA-Seq data. Expression levels were computed by using Rsubread (v1.28) [62]. The information of maize known miRNAs was extracted from miRBase (release 22). Novel miRNAs were predicted by MiRDeep2 (v2.0.0.8) [63], and the miRNA target gene set was the union result predicted by using tapir (v1.2) [64] and psRobot (v1.2) [65]. Transcript sequences predicted by gene models (AGPv4, Ensembl release 37) of the maize WBC/ABCG family genes were used in miRNA target prediction. Default parameters were used in these analyses.

### 4.4. Plasmid Construction and Maize Transformation

For generating CRISPR/Cas9-mediated mutants, the CRISPR/Cas9 plasmids were constructed based on the *pBUE411* vector as described previously [66]. The CRISPR-P 2.0 (http://crispr.hzau.edu.cn/CRISPR2/, accessed on 13 May 2019) was used to choose specific gRNAs targeting the selected genes, and then the off-target analysis of gRNAs sequences was carried out on the website (http://www.rgenome.net/cas-offinder/) (accessed on 13 May 2019). A CRISPR/Cas9 plasmid contains two gRNAs. For assembly of the two gRNAs, a PCR fragment was amplified based on *pCBC-MT1T2* with the primer pair specific for each gene (Table S2) and then purified PCR fragments were cloned into BsaI-digested *pBUE411* vector.

Maize hybrid Hi II was used as the transformation receptor. The CRISPR/Cas9 vector was introduced in *Agrobacterium tumefaciens* strain EHA105 and transformed into immature embryos as described previously [67]. The Bar gene was used as a selectable marker and positive transformants were then selected by PCR amplification using primers OGF41 and OGF42. The primers were listed in Table S2.

### 4.5. Genotyping Maize Plants

Genomic DNA was extracted from leaves of maize seedlings for the T_0_, F_1,_ and F_2_ generations using the cetyltrimethylammonium bromide (CTAB) method [68]. PCR amplifications of relevant regions were performed using the specific primers flanking the target sites listed in Table S2, and then the purified PCR products were cloned into the *pEASY-T1* vector (TransGen Biotech, Beijing, China) for Sanger sequencing. The sequencing chromatograms were carefully examined for exact patterns that might indicate the mutation types, such as homozygous, monoallelic, or diallelic mutations. For genotyping plants from the F_2_ generation, cosegregating molecular markers were designed according to mutations of target genes (Table S2) as described previously [51], and the PCR products were analyzed by agarose gel electrophoresis or polyacrylamide gel electrophoresis.

### 4.6. Quantitative Real-Time PCR (qRT-PCR) Analysis

Total RNA was isolated using TRIzol reagent (Invitrogen, Waltham, MA, USA), and genomic DNA was removed with DNase I (Promega, Madison, WI, USA). The cDNA was synthesized using 5X All-In-One RT MasterMix (abm, Richmond, Canada) following the manufacturer’s protocol. The reverse transcription of microRNA was performed using PrimeScript RT reagent Kit (TaKaRa, Kusatsu, Japan) according to Chen et al. [69]. qRT-PCR was conducted with the corresponding primer set (Table S2) on a QuantStudio 5 Real-Time PCR system (ABI, Waltham, MA, USA) using TB Green Premix EX Tag (TakaRa, Kusatsu, Japan). Both *ZmUbi2* and *ZmCyanase* were used as two internal controls. U6 snRNA was used as the housekeeping RNA in the expression analysis of microRNA. Each sample had three technical replicates and three biological replicates. The amplification data were calculated by the 2^−ΔΔCt^ method [70], and quantitative results were shown as means ± SD.

### 4.7. Phylogenetic Analysis

WBC/ABCG family members in maize, rice, and *Arabidopsis thaliana* were obtained from previous studies [16,71,72]. The phylogenetic tree was reconstructed in MEGA7.0 using the neighbor-joining method, with Poisson correction, pairwise deletion, and 1000 bootstrap replicates.

### 4.8. Subcellular Localization of ZmABCG26 and ZmFAR1

The corresponding full-length cDNAs of *ZmABCG26* and *ZmFAR1* were amplified using the primer pairs listed in Table S2 and fused in-frame with N terminal of GFP and then inserted to the expression vectors *pUC19* and *pJG186* via infusion method, respectively. The ER marker mCherry-HDEL was created by adding the sequence encoding HDEL to the 3′ end of mCherry [73]. The vector of plasma membrane fluorescent marker CFP-AtROP10 was kindly provided by Yule Liu’s Lab [74]. Constructs were transiently expressed in maize protoplasts or tobacco leaves. To detect the GFP- or mCherry-tagged proteins, an LSM780 confocal laser scanning microscope (Zeiss, Jena, Germany) was used.

### 4.9. Enzyme Activity Assay of ZmFAR1 and Its Mutants

Using a basic local alignment searching tool—protein to protein (BLASTP) search on the GRAMENE website (http://www.gramene.org/, accessed on 28 August 2020), the sequences of three orthologs of ZmFAR1 were obtained, and then sequence alignment of these orthologous proteins was performed to predict the conserved motifs (I and II) and active sites [75]. To generate the four mutants of ZmFAR1, the overlap-extension PCR method was performed with specific primers by substitutions of G101A, G104A, Y327F, and K331I. To construct the expression vectors, each of the full-length cDNAs of *ZmFAR1* and the point mutation sequences was fused to the maltose-binding protein (MBP) gene and cloned into the vector *pMAL-C2X* (New England Biolabs, Ipswich, MA, USA). The fusion proteins of ZmFAR1-MBP, G101A-MBP, G104A-MBP, Y327F-MBP, and K331I-MBP were expressed in *E. coli* strain BL21, purified by using Ni affinity column, and then subjected by SDS–PAGE analysis with conformation by Western blot using MBP antibody (New England Biolabs, Ipswich, MA, USA). The primer sequences used for PCR amplification were listed in Table S2.

Enzyme activities of ZmFAR1, G101A, G104A, Y327F, and K331I were determined using lauroyl (C12:0) CoA, palmitoyl (C16:0) CoA, and stearoyl (C18:0) CoA (Sigma, St. Louis, MO, USA) as substrates. The standard reaction system consisted of 20 mM Tris-HCl (pH 7.0), 80 mM NaCl (pH 7.0), 5 µM C12:0 CoA, C16:0 CoA, or C18:0 CoA, 200 µM NADPH, and 0.1–0.2 µM ZmFAR1 or the mutant protein. The reaction was incubated at 30 °C for 10 min, and absorbance was measured at 340 nm to calculate enzyme activities. Substrate specificity analyses of ZmFAR1 and its four mutants were performed at 30 °C for 30 min in 20 mM Tris-HCl (pH 8.0), 80 mM NaCl, and 200 µM NADPH, as well as 5 µM C12:0 CoA, C16:0 CoA, or C18:0 CoA.

### 4.10. Analysis of Anther Wax, Cutin, and Internal Fatty Acids

Stage 13 WT, *zmfar1,* and *zmabcg26* anthers were collected and freeze-dried using an α 2–4 LD plus freeze dryer (Christ, Osterode, Germany). Micrographs were collected to determine the surface area of anthers, assuming a cylindrical body for maize anthers as described previously [37]. Wax, cutin, and internal fatty acids were analyzed as described previously [46].

## Data Availability

All data are shown in the main manuscript and in the Supplementary Materials.

## Supplementary Materials

Supplementary materials can be found at https://www.mdpi.com/article/10.3390/ijms22157916/s1.

## Abbreviations

ABCG	ATP-Binding Cassette Transporter G
ALC	Alcohol
ALK	Alkane
DHDFA	Docosahexaenoic Acid Fatty Acid
FA	Fatty Acid
FAR	Fatty Acyl Reductase
GC–MS	Gas Chromatography–Mass Spectrometry
GMS	Genic Male Sterility
HFA	Hydroxy Fatty Acid
miRNA	microRNA
miRNA-Seq	microRNA Sequencing
PAGE	Polyacrylamide Gel Electrophoresis
qRT-PCR	quantitative Reverse Transcription PCR
RNA-Seq	RNA Sequencing
SEM	Scanning Electron Microscopy
WBC	White Brown Complex
WT	Wild Type

## References

[1] Zhang D., Yang L. (2014). Specification of tapetum and microsporocyte cells within the anther. Curr. Opin. Plant Biol..

[2] Jiang J., Zhang Z., Cao J. (2013). Pollen wall development: The associated enzymes and metabolic pathways. Plant Biol..

[3] Shi J., Cui M., Yang L., Kim Y.J., Zhang D. (2015). Genetic and biochemical mechanisms of pollen wall development. Trends Plant Sci..

[4] Wan X., Wu S., Li Z., An X., Tian Y. (2020). Lipid metabolism: Critical roles in male fertility and other aspects of reproductive development in plants. Mol. Plant.

[5] Ishiguro S., Kawai-Oda A., Ueda J., Nishida I., Okada K. (2001). The DEFECTIVE IN ANTHER DEHISCENCE1 gene encodes a novel phospholipase A1 catalyzing the initial step of jasmonic acid biosynthesis, which synchronizes pollen maturation, anther dehiscence, and flower opening in *Arabidopsis*. Plant Cell.

[6] Xiao Y., Chen Y., Charnikhova T., Mulder P.P., Heijmans J., Hoogenboom A., Agalou A., Michel C., Morel J.B., Dreni L. (2014). *OsJAR1* is required for JA-regulated floret opening and anther dehiscence in rice. Plant Mol. Biol..

[7] Zhu T., Li Z., An X., Long Y., Xue X., Xie K., Ma B., Zhang D., Guan Y., Niu C. (2020). Normal structure and function of endothecium chloroplasts maintained by ZmMs33-mediated lipid biosynthesis in tapetal cells are critical for anther development in maize. Mol. Plant.

[8] Wang J., Bao J.L., Zhou B.B., Li M., Li X.Z., Jin J. (2021). The *osa-miR164* target *OsCUC1* functions redundantly with *OsCUC3* in controlling rice meristem/organ boundary specification. New Phytol..

[9] Millar A.A., Lohe A., Wong G. (2019). Biology and function of miR159 in plants. Plants.

[10] Wang H., Mao Y., Yang J., He Y. (2015). *TCP24* modulates secondary cell wall thickening and anther endothecium development. Front. Plant Sci..

[11] Zheng L., Nagpal P., Villarino G., Trinidad B., Bird L., Huang Y., Reed J.W. (2019). miR167 limits anther growth to potentiate anther dehiscence. Development.

[12] Li Z., Zhu T., Liu S., Jiang Y., Liu H., Zhang Y., Xie K., Li J., An X., Wan X. (2021). Genome-wide analyses on transcription factors and their potential microRNA regulators involved in maize male fertility. Crop J..

[13] Zhang Y., He R., Lian J., Zhou Y., Zhang F., Li Q., Yu Y., Feng Y., Yang Y., Lei M. (2020). OsmiR528 regulates rice-pollen intine formation by targeting an uclacyanin to influence flavonoid metabolism. Proc. Natl. Acad. Sci. USA.

[14] Wang R., Fang Y., Wu X., Qing M., Li C., Xie K., Deng X., Guo W. (2020). The miR399-*CsUBC24* module regulates reproductive development and male fertility in citrus. Plant Physiol..

[15] Verrier P.J., Bird D., Buria B., Dassa E., Forestier C., Geisler M., Klein M., Kolukisaoglu U., Lee Y., Martinoia E. (2008). Plant ABC proteins–a unified nomenclature and updated inventory. Trends Plant Sci..

[16] Pang K., Li Y., Liu M., Meng Z., Yu Y. (2013). Inventory and general analysis of the ATP-binding cassette (ABC) gene superfamily in maize (*Zea mays* L.). Gene.

[17] Bessire M., Borel S., Fabre G., Carraca L., Efremova N., Yephremov A., Cao Y., Jetter R., Jacquat A.-C., Metraux J.-P. (2011). A member of the PLEIOTROPIC DRUG RESISTANCE family of ATP binding cassette transporters is required for the formation of a functional cuticle in *Arabidopsis*. Plant Cell.

[18] Choi H., Jin J.Y., Choi S., Hwang J.U., Kim Y.Y., Suh M.C., Lee Y. (2011). An ABCG/WBC-type ABC transporter is essential for transport of sporopollenin precursors for exine formation in developing pollen. Plant J..

[19] Quilichini T.D., Friedmann M.C., Samuels A.L., Douglas C.J. (2010). ATP-binding cassette transporter G26 is required for male fertility and pollen exine formation in *Arabidopsis*. Plant Physiol..

[20] Yadav V., Molina I., Ranathunge K., Castillo I.Q., Rothstein S.J., Reed J.W. (2014). ABCG transporters are required for suberin and pollen wall extracellular barriers in *Arabidopsis*. Plant Cell.

[21] Choi H., Ohyama K., Kim Y.-Y., Jin J.-Y., Lee S.B., Yamaoka Y., Muranaka T., Suh M.C., Fujioka S., Lee Y. (2014). The role of *Arabidopsis* ABCG9 and ABCG31 ATP binding cassette transporters in pollen fitness and the deposition of steryl glycosides on the pollen coat. Plant Cell.

[22] Panikashvili D., Shi J.X., Schreiber L., Aharoni A. (2011). The *Arabidopsis* ABCG13 transporter is required for flower cuticle secretion and patterning of the petal epidermis. New Phytol..

[23] McFarlane H.E., Shin J.J., Bird D.A., Samuels A.L. (2010). *Arabidopsis* ABCG transporters, which are required for export of diverse cuticular lipids, dimerize in different combinations. Plant Cell.

[24] Panikashvili D., Shi J.X., Bocobza S., Franke R.B., Schreiber L., Aharoni A. (2010). The *Arabidopsis* DSO/ABCG11 transporter affects cutin metabolism in reproductive organs and suberin in roots. Mol. Plant.

[25] Zhu B.T., Li H., Xia X.Z., Meng Y.Y., Wang N., Li L.L., Shi J.X., Pei Y.X., Lin M., Niu L.F. (2020). ATP-binding cassette G transporters SGE1 and MtABCG13 control stigma exsertion. Plant Physiol..

[26] Niu B.X., He F.R., He M., Ren D., Chen L.T., Liu Y.G. (2013). The ATP-binding cassette transporter OsABCG15 is required for anther development and pollen fertility in rice. J. Integr. Plant Biol..

[27] Zhao G., Shi J., Liang W., Xue F., Luo Q., Zhu L., Qu G., Chen M., Schreiber L., Zhang D. (2015). Two ATP binding cassette G transporters, rice ATP binding cassette G26 and ATP binding cassette G15, collaboratively regulate rice male reproduction. Plant Physiol..

[28] Chang Z., Jin M., Yan W., Chen H., Qiu S., Fu S., Xia J., Liu Y., Chen Z., Wu J. (2018). The ATP-binding cassette (ABC) transporter OsABCG3 is essential for pollen development in rice. Rice.

[29] Luo T., Zou T., Yuan G.Q., He Z.Y., Li W.J., Tao Y., Liu M.M., Zhou D., Zhao H.F., Zhu J. (2020). *Less and shrunken pollen 1* (LSP1) encodes a member of the ABC transporter family required for pollen wall development in rice (*Oryza sativa* L.). Crop J..

[30] Li L., Li D.L., Liu S.Z., Ma X.L., Dietrich C.R., Hu H.C., Zhang G.S., Liu Z.Y., Zheng J., Wang G.Y. (2013). The Maize *glossy13* Gene, cloned via BSR-Seq and Seq-Walking encodes a putative ABC transporter required for the normal accumulation of epicuticular waxes. PLoS ONE.

[31] Xu Q., Yang L., Kang D., Ren Z., Liu Y. (2021). Maize *MS2* encodes an ATP-binding cassette transporter that is essential for anther development. Crop J..

[32] Kunst L., Samuels A.L. (2003). Biosynthesis and secretion of plant cuticular wax. Prog. Lipid Res..

[33] Aarts M., Hodge R., Kalantidis K., Florack D., Wilson Z., Mulligan B., Stiekema W., Scott R., Pereira A. (1997). The *Arabidopsis* MALE STERILITY 2 protein shares similarity with reductases in elongation/condensation complexes. Plant J..

[34] Chen W., Yu X.H., Zhang K., Shi J., De Oliveira S., Schreiber L., Shanklin J., Zhang D. (2011). *Male Sterile2* encodes a plastid-localized fatty acyl carrier protein reductase required for pollen exine development in *Arabidopsis*. Plant Physiol..

[35] Wang K., Guo Z., Zhou W., Zhang C., Zhang Z., Lou Y., Xiong S., Yao X., Fan J., Zhu J. (2018). The regulation of sporopollenin biosynthesis genes for rapid pollen wall formation. Plant Physiol..

[36] Rowland O., Zheng H., Hepworth S.R., Lam P., Jetter R., Kunst L. (2006). CER4 encodes an alcohol-forming fatty acyl-coenzyme A reductase involved in cuticular wax production in Arabidopsis. Plant Physiol..

[37] Shi J., Tan H., Yu X.H., Liu Y., Liang W., Ranathunge K., Franke R.B., Schreiber L., Wang Y., Kai G. (2011). *Defective pollen wall* is required for anther and microspore development in rice and encodes a fatty acyl carrier protein reductase. Plant Cell.

[38] Pan X., Yan W., Chang Z., Xu Y., Luo M., Xu C., Chen Z., Wu J., Tang X. (2020). OsMYB80 regulates anther development and pollen fertility by targeting multiple biological pathways. Plant Cell Physiol..

[39] Wang A.M., Xia Q., Xie W.S., Dumonceaux T., Zou J.T., Datla R., Selvaraj G. (2002). Male gametophyte development in bread wheat (*Triticum aestivum* L.): Molecular, cellular, and biochemical analyses of a sporophytic contribution to pollen wall ontogeny. Plant J..

[40] Tian Y., Xiao S., Liu J., Somaratne Y., Zhang H., Wang M., Zhang H., Zhao L., Chen H. (2017). *MALE STERILE6021* (*MS6021*) is required for the development of anther cuticle and pollen exine in maize. Sci. Rep..

[41] Zhang S., Wu S., Niu C., Liu D., Yan T., Tian Y., Liu S., Xie K., Li Z., Wang Y. (2021). *ZmMs25* encoding a plastid-localized fatty acyl reductase is critical for anther and pollen development in maize. J. Exp. Bot..

[42] Li Z., An X., Zhu T., Yan T., Wu S., Tian Y., Li J., Wan X. (2019). Discovering and constructing ceRNA-miRNA-target gene regulatory networks during anther development in maize. Int. J. Mol. Sci..

[43] Zhai J., Zhang H., Arikit S., Huang K., Nan G.-L., Walbot V., Meyers B.C. (2015). Spatiotemporally dynamic, cell-type-dependent premeiotic and meiotic phasiRNAs in maize anthers. Proc. Natl. Acad. Sci. USA.

[44] An X., Ma B., Duan M., Dong Z., Liu R., Yuan D., Hou Q., Wu S., Zhang D., Liu D. (2020). Molecular regulation of *ZmMs7* required for maize male fertility and development of a dominant male-sterility system in multiple species. Proc. Natl. Acad. Sci. USA.

[45] Zhang D., Wu S., An X., Xie K., Dong Z., Zhou Y., Xu L., Fang W., Liu S., Liu S. (2018). Construction of a multicontrol sterility system for a maize male-sterile line and hybrid seed production based on the *ZmMs7* gene encoding a PHD-finger transcription factor. Plant Biotechnol. J..

[46] An X., Dong Z., Tian Y., Xie K., Wu S., Zhu T., Zhang D., Zhou Y., Niu C., Ma B. (2019). *ZmMs30* encoding a novel GDSL lipase is essential for male fertility and valuable for hybrid breeding in maize. Mol. Plant.

[47] Xie K., Wu S., Li Z., Zhou Y., Zhang D., Dong Z., An X., Zhu T., Zhang S., Liu S. (2018). Map-based cloning and characterization of *Zea mays male sterility33* (*ZmMs33*) gene, encoding a glycerol-3-phosphate acyltransferase. Theor. Appl. Genet..

[48] Zhu T., Wu S., Zhang D., Li Z., Xie K., An X., Ma B., Hou Q., Dong Z., Tian Y. (2019). Genome-wide analysis of maize GPAT gene family and cytological characterization and breeding application of *ZmMs33*/*ZmGPAT6* gene. Theor. Appl. Genet..

[49] Somaratne Y., Tian Y., Zhang H., Wang M., Huo Y., Cao F., Zhao L., Chen H. (2017). ABNORMAL POLLEN VACUOLATION1 (APV1) is required for male fertility by contributing to anther cuticle and pollen exine formation in maize. Plant J..

[50] Chen X., Zhang H., Sun H., Luo H., Zhao L., Dong Z., Yan S., Zhao C., Liu R., Xu C. (2017). IRREGULAR POLLEN EXINE1 is a novel factor in anther cuticle and pollen exine formation. Plant Physiol..

[51] Wang Y., Liu D., Tian Y., Wu S., An X., Dong Z., Zhang S., Bao J., Li Z., Li J. (2019). Map-based cloning, phylogenetic, and microsynteny analyses of *ZmMs20* gene regulating male fertility in maize. Int. J. Mol. Sci..

[52] Djukanovic V., Smith J., Lowe K., Yang M., Gao H., Jones S., Nicholson M.G., West A., Lape J., Bidney D. (2013). Male-sterile maize plants produced by targeted mutagenesis of the cytochrome P450-like gene (*MS26*) using a re-designed I-*CreI* homing endonuclease. Plant J..

[53] Yang X., Wu D., Shi J., He Y., Pinot F., Grausem B., Yin C., Zhu L., Chen M., Luo Z. (2014). Rice CYP703A3, a cytochrome P450 hydroxylase, is essential for development of anther cuticle and pollen exine. J. Integra. Plant Biol..

[54] Li H., Pinot F., Sauveplane V., Werck-Reichhart D., Diehl P., Schreiber L., Franke R., Zhang P., Chen L., Gao Y. (2010). Cytochrome P450 family member CYP704B2 catalyzes the omega-hydroxylation of fatty acids and is required for anther cutin biosynthesis and pollen exine formation in rice. Plant Cell.

[55] Jung K.H., Han M.J., Lee D.Y., Lee Y.S., Schreiber L., Franke R., Faust A., Yephremov A., Saedler H., Kim Y.W. (2006). *Wax-deficient anther1* is involved in cuticle and wax production in rice anther walls and is required for pollen development. Plant Cell.

[56] Kurdyukov S., Faust A., Nawrath C., Bar S., Voisin D., Efremova N., Franke R., Schreiber L., Saedler H., Metraux J.P. (2006). The epidermis-specific extracellular BODYGUARD controls cuticle development and morphogenesis in *Arabidopsis*. Plant Cell.

[57] Patel R.K., Jain M. (2012). NGS QC toolkit: A toolkit for quality control of next generation sequencing data. PLoS ONE.

[58] Nawrocki E.P., Eddy S.R. (2013). Infernal 1.1: 100-fold faster RNA homology searches. Bioinformatics.

[59] Kalvari I., Argasinska J., Quinones-Olvera N., Nawrocki E.P., Rivas E., Eddy S.R., Bateman A., Finn R.D., Petrov A.I. (2018). Rfam 13.0: Shifting to a genome-centric resource for non-coding RNA families. Nucleic. Acids Res..

[60] Trapnell C., Pachter L., Salzberg S.L. (2009). TopHat: Discovering splice junctions with RNA-Seq. Bioinformatics.

[61] Langmead B., Trapnell C., Pop M., Salzberg S.L. (2009). Ultrafast and memory-efficient alignment of short DNA sequences to the human genome. Genome Biol..

[62] Liao Y., Smyth G.K., Shi W. (2019). The R package Rsubread is easier, faster, cheaper and better for alignment and quantification of RNA sequencing reads. Nucleic. Acids Res..

[63] Friedlaender M.R., Mackowiak S.D., Li N., Chen W., Rajewsky N. (2012). miRDeep2 accurately identifies known and hundreds of novel microRNA genes in seven animal clades. Nucleic. Acids Res..

[64] Bonnet E., He Y., Billiau K., Van de Peer Y. (2010). TAPIR, a web server for the prediction of plant microRNA targets, including target mimics. Bioinformatics.

[65] Wu H.-J., Ma Y.-K., Chen T., Wang M., Wang X.-J. (2012). PsRobot: A web-based plant small RNA meta-analysis toolbox. Nucleic. Acids Res..

[66] Xing H.L., Dong L., Wang Z.P., Zhang H.Y., Han C.Y., Liu B., Wang X.C., Chen Q.J. (2014). A CRISPR/Cas9 toolkit for multiplex genome editing in plants. BMC Plant Biol..

[67] Frame B.R., Shou H., Chikwamba R.K., Zhang Z., Xiang C., Fonger T.M., Pegg S.E., Li B., Nettleton D.S., Pei D. (2002). *Agrobacterium tumefaciens*-mediated transformation of maize embryos using a standard binary vector system. Plant Physiol..

[68] Murray M.G., Thompson W.F. (1980). Rapid isolation of high molecular weight plant DNA. Nucleic. Acids Res..

[69] Chen C., Ridzon D.A., Broomer A.J., Zhou Z., Lee D.H., Nguyen J.T., Barbisin M., Xu N.L., Mahuvakar V.R., Andersen M.R. (2005). Real-time quantification of microRNAs by stem-loop RT-PCR. Nucleic. Acids Res..

[70] Livak K.J., Schmittgen T.D. (2001). Analysis of relative gene expression data using real-time quantitative PCR and the 2^−∆∆^^C_T_^ Method. Methods.

[71] Gupta B.B., Selter L.L., Baranwal V.K., Arora D., Mishra S.K., Sirohi P., Poonia A.K., Chaudharya R., Kumar R., Krattinger S.G. (2019). Updated inventory, evolutionary and expression analyses of G, (PDR) type ABC transporter genes of rice. Plant Physiol. Bioch..

[72] Grafe K., Schmitt L. (2021). The ABC transporter G subfamily in *Arabidopsis thaliana*. J. Exp. Bot..

[73] Nelson B.K., Cai X., Nebenführ A. (2007). A multicolored set of in vivo organelle markers for co-localization studies in *Arabidopsis* and other plants. Plant J..

[74] Chen T.Y., Liu D., Niu X.L., Wang J.Z., Qian L.C., Han L., Liu N., Zhao J.P., Hong Y.G., Liu Y.L. (2017). Antiviral resistance protein Tm-2(2) functions on the plasma membrane. Plant Physiol..

[75] Chacon M.G., Fournier A.E., Tran F., Dittrich-Domergue F., Pulsifer I.P., Domergue F., Rowland O. (2013). Identification of amino acids conferring chain length substrate specificities on fatty alcohol-forming reductases FAR5 and FAR8 from *Arabidopsis thaliana*. J. Biol. Chem..

