# Supplementary material for: ZmFAR1 and ZmABCG26 Regulated by microRNA Are Essential for Lipid Metabolism in Maize Anther
Source: Int J Mol Sci. 2021 Jul 24;22(15):7916. doi: 10.3390/ijms22157916 (PMC8348775; doi:10.3390/ijms22157916)
Supplement: Supplementary file 1 [file ijms-22-07916-s001.zip › Supplementary Materials -20120719/Supplementary Figures-20210718.pdf]

International Journal of
Molecular Sciences

MDPI

Supplymentary Figures

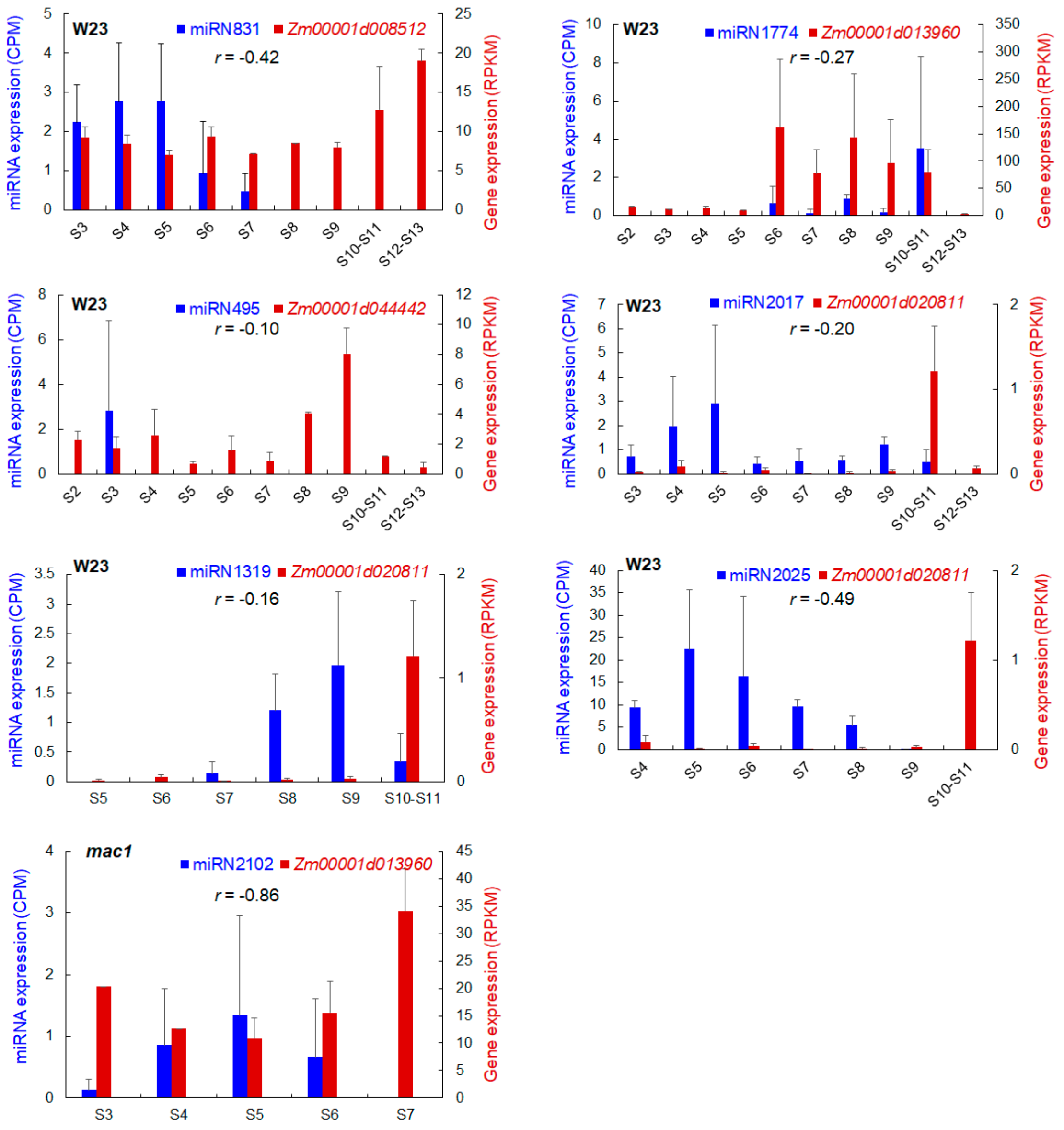

Supplementary Figure 1. The negatively correlated expression patterns of seven miRNA-ABCG gene pairs during maize anther development in transcriptome data. The names of these miRNAs were marked in blue in Figure 1B. Pearson correlation test was used. *r*, Pearson correlation coefficient.

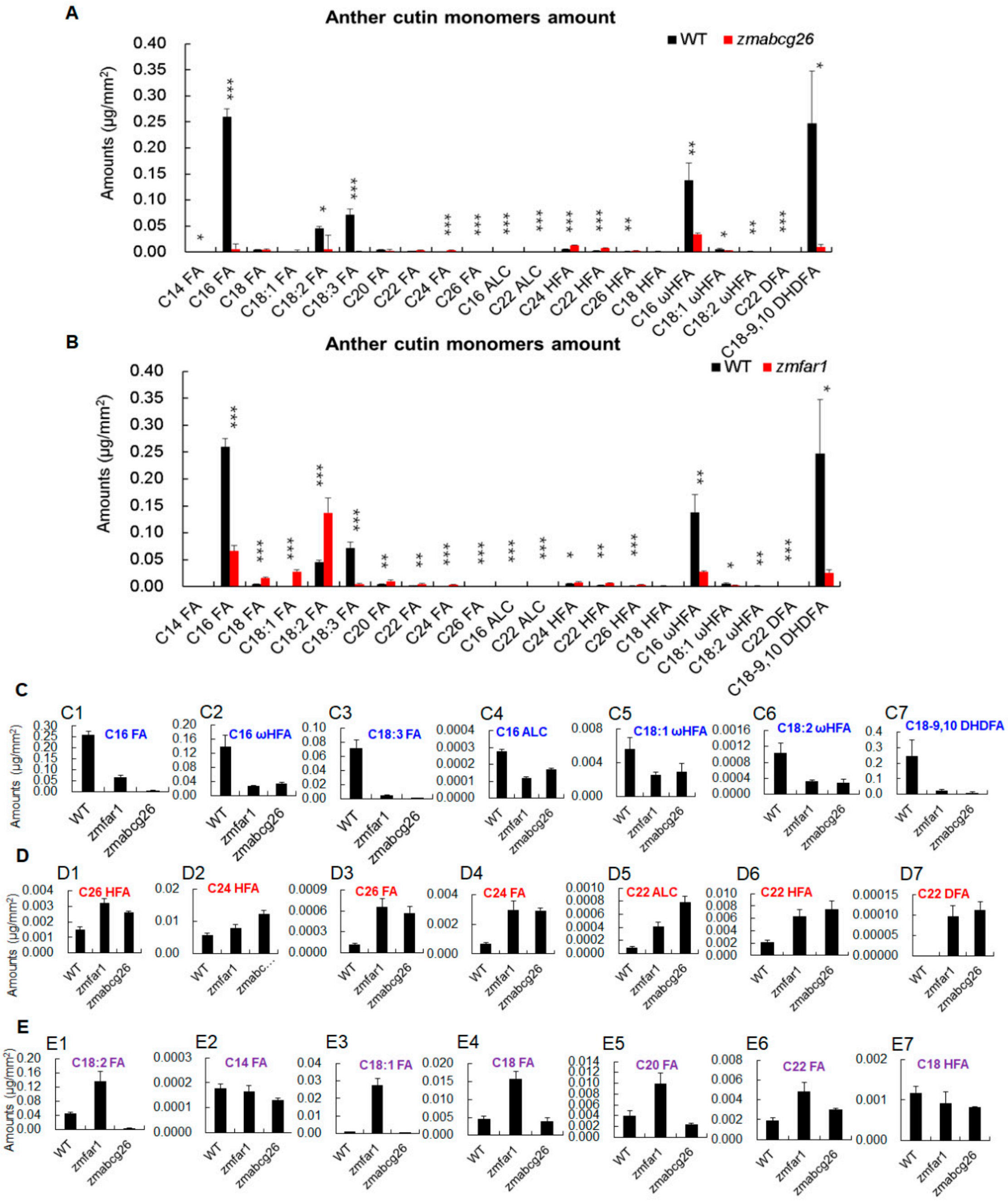

Supplementary Figure 2. The amount changes of cutin monomers in WT and *zmacbg26* and *zmfar1* mutant anthers by lipidomics analysis. (**A**) The amounts of 21 cutin monomers per unit surface area in WT and *zmabcg26* anthers at stage 13. (**B**) The amounts of 21 cutin monomers per unit surface area in WT and *zmfar1* anthers at stage 13. *, ** and *** indicate the significant levels of 5%, 1% and 1‰ (Student's *t* test, n=3), respectively. (**C**) The seven cutin monomers reduced in amounts in both *zmfar1* and *zmabcg26* anthers compared to those in WT at stage 13. (**D**) The seven cutin monomers increased in amounts in both *zmfar1* and *zmabcg26* anthers compared to those in WT at stage 13. (**E**) The seven cutin monomers oppositely or differently changed in amounts in *zmfar1* and *zmabcg26* anthers compared to those in WT at stage 13.

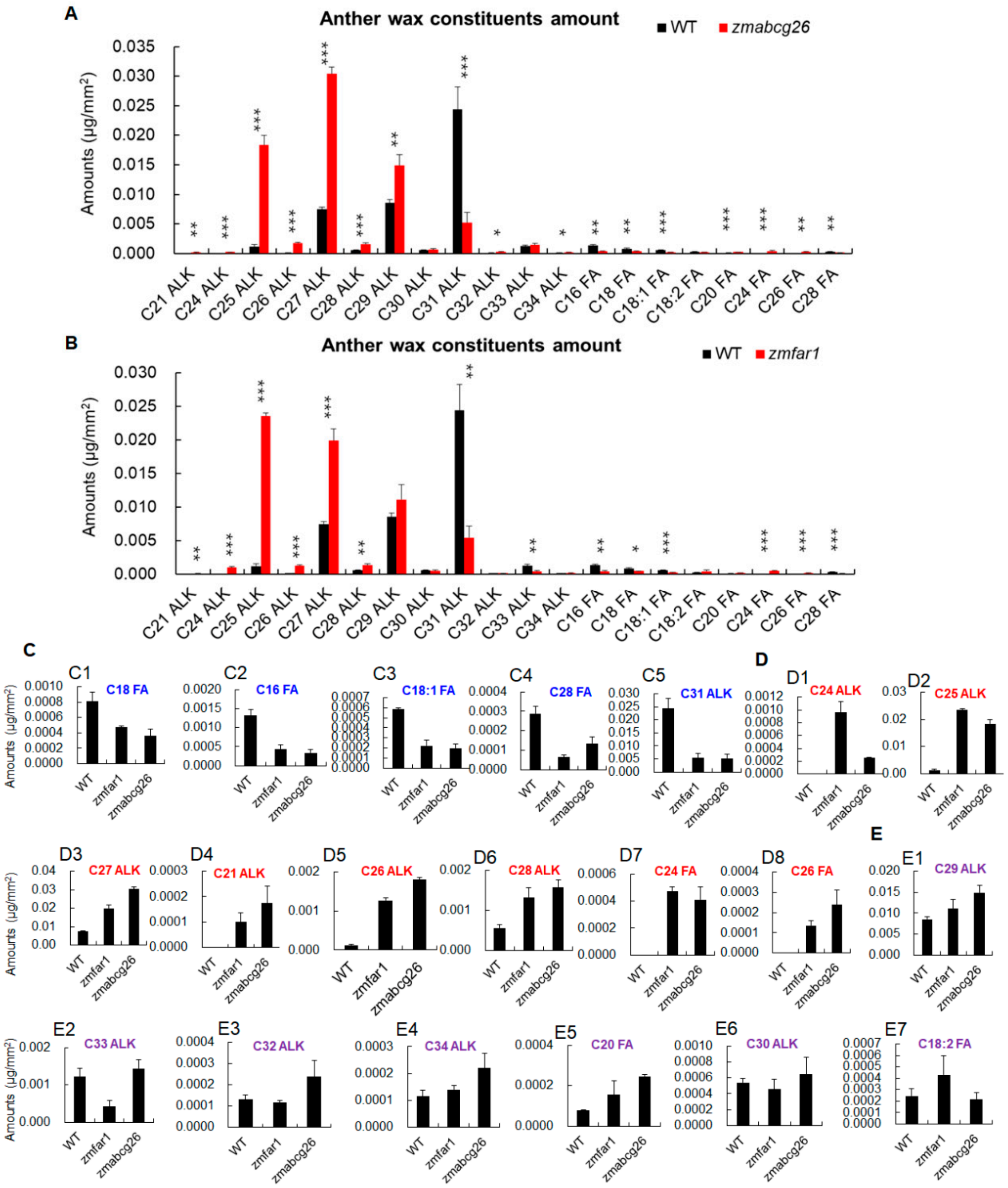

Supplementary Figure 3. The amount changes of wax constituents in WT and *zmacbg26* and *zmfar1* mutant anthers by lipidomics analysis. (**A**) The amounts of 20 wax constituents per unit surface area in WT and *zmabcg26* anthers at stage 13. (**B**) The amounts of wax constituents per unit surface area in WT and *zmfar1* anthers at stage 13. *, ** and *** indicate the significant levels of 5%, 1% and 1‰ (Student's *t* test, n=3), respectively. (**C**) The five wax constituents reduced in amounts in both *zmfar1* and *zmabcg26* anthers compared to those in WT at stage 13. (**D**) The eight wax constituents increased in amounts in both *zmfar1* and *zmabcg26* anthers compared to those in WT at stage 13. (**E**) The seven wax constituents oppositely or differently changed in amounts in *zmfar1* and *zmabcg26* anthers compared to those in WT at stage 13.

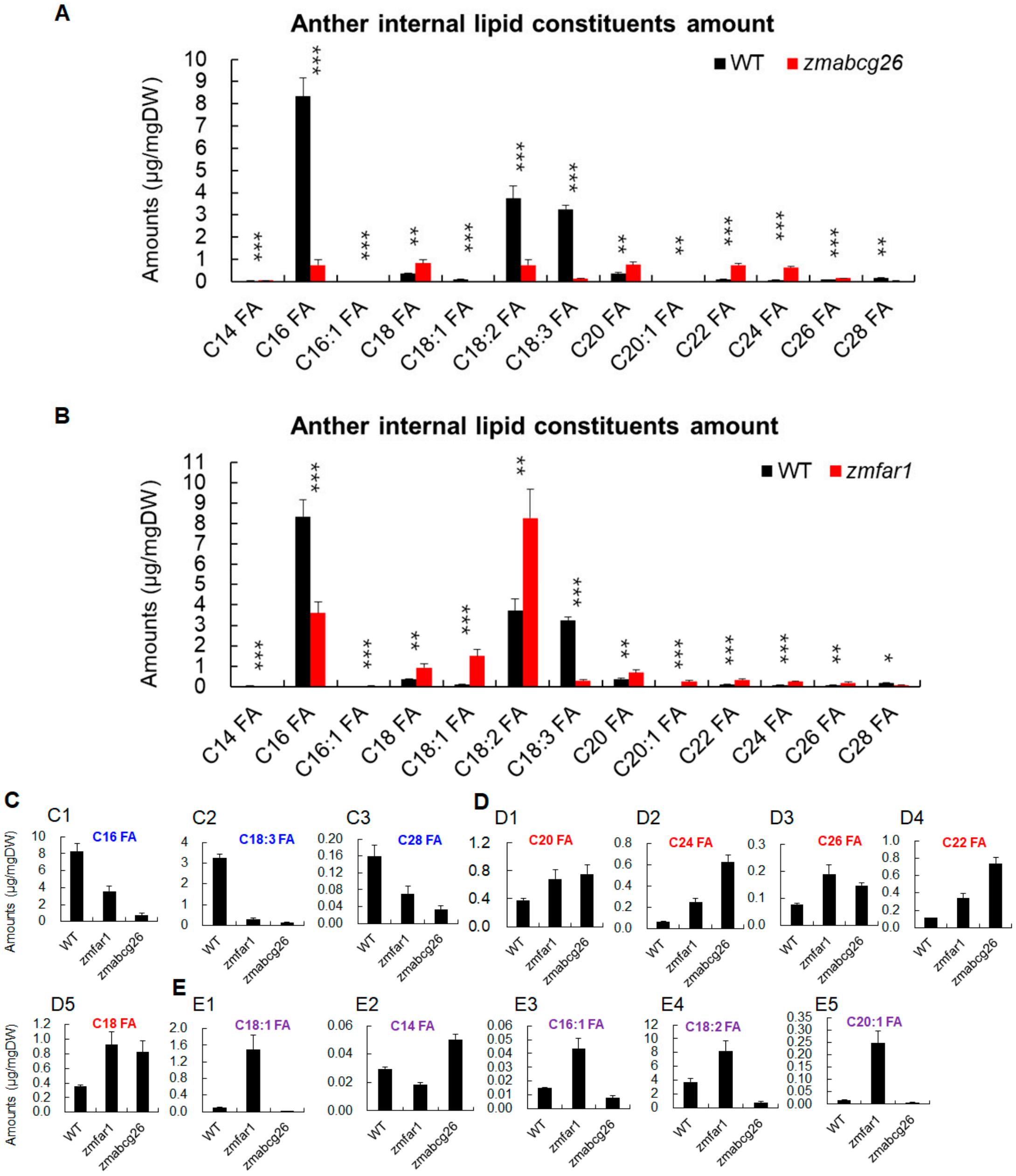

Supplementary Figure 4. The amount changes of internal lipid constituents in WT and *zmacbg26* and *zmfar1* mutant anthers by lipidomics analysis. (**A**) The amounts of 13 internal lipid constituents per unit dry weight in WT and *zmabcg26* anthers at stage 13. (**B**) The amounts of 13 internal lipid constituents per unit dry weight in WT and *zmfar1* anthers at stage 13. *, ** and *** indicate the significant levels of 5%, 1% and 1‰ (Student's *t* test, n=3), respectively. (**C**) The three internal lipid constituents reduced in amounts in both *zmfar1* and *zmabcg26* anthers at stage 13 compared to those in WT. (**D**) The five internal lipid constituents increased in amounts in both *zmfar1* and *zmabcg26* anthers at stage 13 compared to those in WT. (**E**) The five internal lipid constituents oppositely or differently changed in amounts in *zmfar1* and *zmabcg26* anthers at stage 13 compared to those in WT.

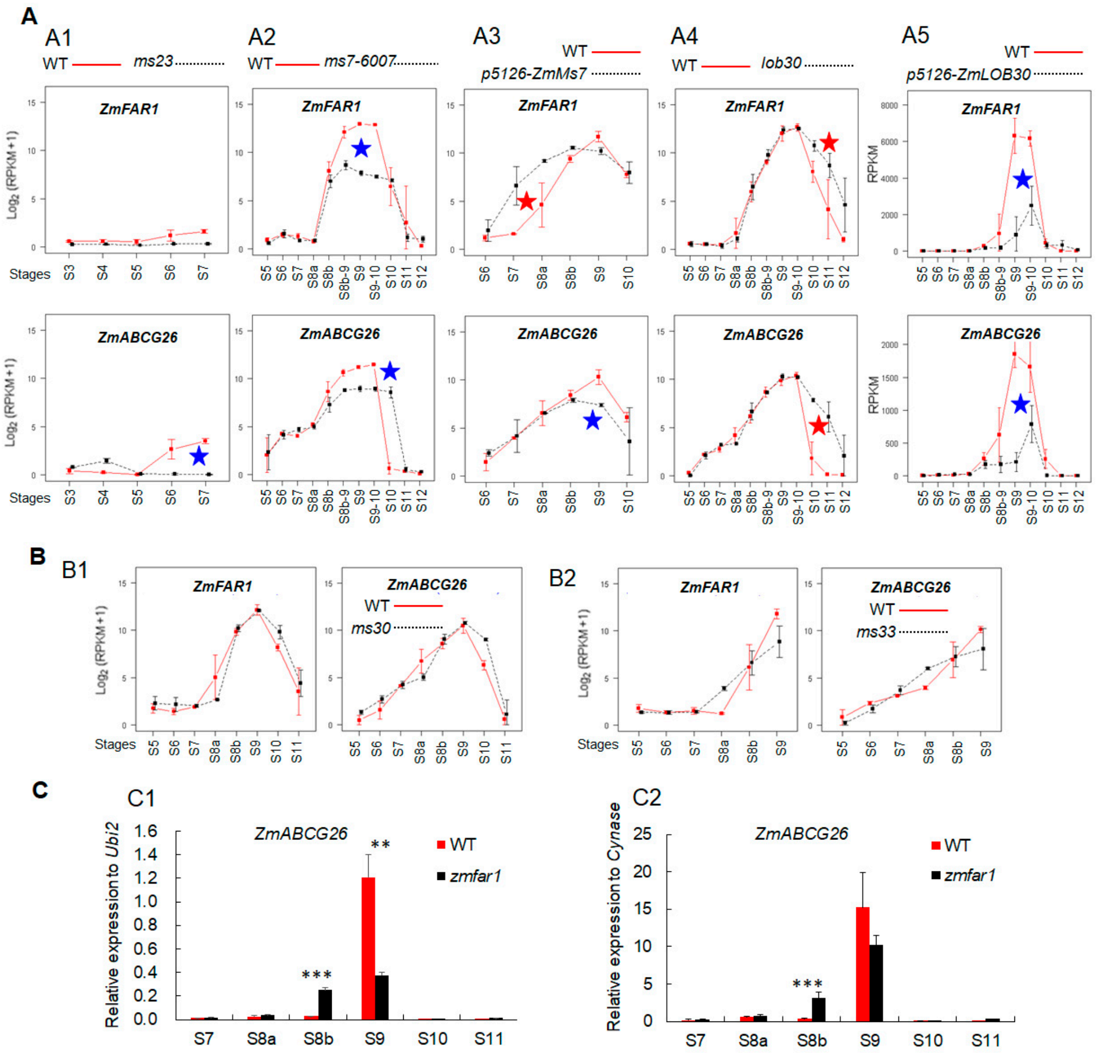

Supplementary Figure 5. The expression pattern changes of *ZmFAR1* and *ZmABCG26* in anthers between WT and GMS mutants or WT and GMS gene over-expression lines by transcriptome and qRT-PCR analyses. (**A**) Transcriptome data of maize anthers at multiple developmental stages from *ms23* (**A1**), *ms7-6007* (**A2**), *p5126-ZmMs7* (**A3**; *ZmMs7*-overexpression line), *lob30* (**A4**), *p5126-ZmLOB30* (**A5**; *ZmLOB30*-overexpression line) mutants or overexpression lines. *ZmMs23*, *ZmMs7* and *ZmLOB30* encoded transcription factors. Genes with up-regulation or down-regulation expressions in mutants or over-expression lines compared with their corresponding WT lines were marked with red or blue stars, respectively. (**B**) Transcriptome data of maize anthers at multiple developmental stages from *ms30* (**B1**) and *ms33* (**B2**) mutants lines. *ZmMs30* and *ZmMs33* encoded proteins related to lipid metabolism in maize anthers. *ZmFAR1* and *ZmABCG26* were not found to be significantly differential expressions between WT and *ms30* or *ms33*. (**C**) Expression pattern changes of *ZmABCG26* between WT and *zmfar1* mutant anthers from stages 7 to 11 using qRT-PCR assay. Maize *ZmUbi2* (**C1**) and *ZmCyanase* (**C2**) genes were used as the internal normalization controls, respectively. ** and *** indicate the significant levels of 1% and 1‰ (Student's *t* test, n=3), respectively.
